# Clinical development of mRNA therapies against solid tumors

**DOI:** 10.1186/s13045-023-01457-x

**Published:** 2023-07-18

**Authors:** Dawei Wu, Lingfeng Hu, Xin Wang, Yue Yu, Shuo-Peng Jia, Hui-Yao Huang, Zi-Wei Li, Jin-Feng Ma, Hai-Bo Zhu, Yu Tang, Ning Li

**Affiliations:** 1 Clinical Trials Center, National Cancer Center/National Clinical Research Center for Cancer/Cancer Hospital, Chinese Academy of Medical Sciences and Peking Union Medical College, Beijing, China; 2 Institute of Medical Biology, Chinese Academy of Medical Sciences and Peking Union Medical College, Kunming, China; 3 Department of Clinical Trials Center, Shanxi Province Cancer Hospital/Shanxi Hospital Affiliated to Cancer Hospital, Chinese Academy of Medical Sciences/Cancer Hospital Affiliated to Shanxi Medical University, Taiyuan, China; 4 Department of Clinical Trials Center, National Cancer Center/National Clinical Research Center for Cancer/Hebei Cancer Hospital, Chinese Academy of Medical Sciences, Langfang, China; 5 Department of Basic Medicine and Clinical Pharmacy, China Pharmaceutical University, Nanjing, China

**Keywords:** Solid tumor, Messenger RNA, Clinical trial, Delivery system, Tumor antigen

## Abstract

The mRNA-based therapeutics have become the hot spot of biopharmaceutical industries in recent years. The landscape of this area is expanding from infectious disease to cancer, which needs to be summarized to provide data supports for industries and research institutions. Based on the Trialtrove database, a total of 108 clinical trials from 1999 to 2021 were retrospectively analyzed. We have demonstrated that the clinical development of mRNA therapies against solid tumors is still at an early stage. There are evolutions in delivery systems from the dendritic cell to the lipid-based platform and in encoding strategies from the fixed tumor antigens to the personalized neoantigens. The adjuvant or maintenance therapy and the combination treatment with checkpoint inhibitors are becoming the major clinical development orientation.

**Supplementary Information:**

The online version contains supplementary material available at 10.1186/s13045-023-01457-x.

## To the editor

Since the clinical application of COVID-19 vaccines, messenger RNA (mRNA)-based therapeutics have become the hot spot of biopharmaceutical industries in recent years [1]. With the advantages of personalized preparation, fast production and good immunogenicity [1–3], therapeutic areas of mRNA are expanding to cancer. Dozens of clinical trials have been launched with preliminary results [1–3]. However, there is no evidence of data on the panorama worldwide. Here, we summarize the current progress, put forward suggestions for future clinical development and provide data supports for industries and research institutions.

Based on the Trialtrove database [4], a total of 108 clinical trials for mRNA therapies against solid tumors were identified worldwide by the cut-off date December 31, 2021 (Additional file 1: Fig. S1). Most of the trials were at phase I (79, 73.1%) (Table 1). The exploratory investigator-initiated trials (IITs) accounted for a large proportion (49, 45.4%), and the sponsors were highly concentrated in a few countries (Additional file 1: Table S1), which suggested that the clinical development in this area was still at its early stage. As a result, many challenges and uncertainties have been raised, including how to optimize the delivery systems and encoded proteins of mRNA sequences and how to select clinical scenario.

**Table 1:** Distribution of cancer types by study phase of clinical trials for anticancer mRNA therapeutics

Cancer type	Phase I	Phase II	Phase III	Total
Melanoma	16	6	1	23
Unspecific solid tumors	21	1	0	22
Renal cell carcinoma	6	5	1	12
Prostate cancer	7	2	0	9
Non-small cell lung cancer	6	3	0	9
Glioblastoma	5	4	0	9
Breast cancer	5	0	0	5
Ovarian cancer	2	1	0	3
Hepatocellular carcinoma	2	0	0	2
Head and neck squamous cell carcinoma	1	1	0	2
Colorectal cancer	1	1	0	2
Breast cancer and melanoma	2	0	0	2
Acute myeloid leukemia	2	0	0	2
Pancreatic cancer	1	0	0	1
Multiple myeloma	1	0	0	1
Gastric cancer	0	1	0	1
Esophageal and non-small cell lung cancer	0	1	0	1
Chronic lymphocytic leukemia	1	0	0	1
Bladder cancer	0	1	0	1
Total	79	27	2	108

**Fig. 1:**
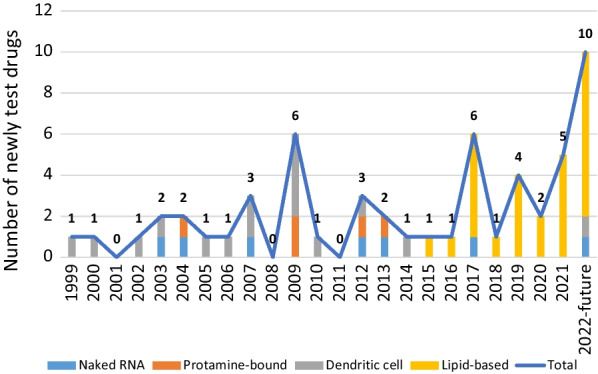
Annual numbers of newly tested mRNA therapeutics by delivery system

A total of 57 mRNA agents were further identified. Analyzing the delivery systems, the lipid-based platform, namely lipid nanoparticle (LNP) (13, 22.8%), lipoplex (LPX) (8, 14.0%) and lipopolyplex (LPP) (6, 10.5%), in aggregate accounted for the largest proportion (27, 47.4%) (Fig. 1, Additional file 1: Table S2). The dendritic cell (DC) was the main platform for drugs developed from 1999 to 2014 (15/25, 60.0%) (Fig. 1), but the ex vivo manipulation of DCs is laborious and time-consuming, with an unsatisfied mRNA transfection rate [1, 2, 5]. Compared with DCs, the lipid-based delivery systems are capable of rapid manufacture, with high plasticity, transfection rate and immunogenicity [6]. Thus, this technology has become the mainstream since 2015 (27/30, 90.0%) (Fig. 1). Given the complexity of the molecular structure, further optimizing the manufacturing process and reducing the unpredictable clinical effects will be the future direction of the lipid-based platform.

The tumor antigen is the most common coding category of mRNA therapies. There were 27 (47.4%), 8 (14.0%) and 5 (8.8%) agents encoding fixed tumor antigens, neoantigens and autologous tumor cell antigens, respectively (Additional file 1: Fig. S2a). MAGE-A1/A3 (6, 10.5%), survivin (6, 10.5%) and tyrosinase (5, 8.8%) were the most common specific encoded proteins (Additional file 1: Fig. S2b), which in line with the features of the most extensively studied cancer types, such as melanoma [7] (Table 1). However, due to the insufficient immunogenicity and the space–time heterogeneity of classic tumor antigens [8], mRNA agents encoding fixed, single antigens have not shown definite clinical benefit yet (Additional file 1: Table S3).

The personalized neoantigen-based therapies are expected to overcome the obstacles. These highly individualized and flexible products perfectly fit the fast manufacture of mRNA, with the development of bioinformatics technology [8]. Since 2009, the number of newly initiated trials for neoantigen-based agents has shown a trend to exceed that of fixed antigens (Additional file 1: Fig. S3). The representatives, mRNA-4157 and BNT-122, have already achieved promising short-term efficacy (Additional file 1: Table S3), though the reliability of the neoantigen prediction system and the encoding capacity of the mRNA sequences for multiple antigens still need to be investigated [9].

Among the 108 trials, there were 32 (29.6%) for adjuvant or maintenance therapy and 59 (54.6%) involving combination treatment, where the immunotherapy was the most prevalent combination strategies (44/59, 74.8%) (Additional file 1: Table S4). The above proportions are relatively high compared with the entire landscape of immuno-oncology [10, 11], which infer that mRNA agents may act as an “assistant” rather than the “backbone” in the cancer treatment. In addition, referring to the rationale of immune checkpoint inhibitors (ICIs), mRNA therapies encoding tumor antigens are more likely to play a greater role in the earlier stage of cancer or advanced stage with lower tumor burden and produce greater synergy with ICIs [12]. Therefore, adjuvant and combination therapies are the trends in the clinical scenarios for mRNA therapies.

In conclusion, the clinical development of mRNA therapies against solid tumors is still at an early stage. The notable shifts in delivery systems from the DC to the lipid-based platform and in encoding strategies from the fixed tumor antigens to the personalized neoantigens together mark a new era in this field. The adjuvant or maintenance therapy and the combination treatment with ICIs are becoming the important clinical development orientation.

## Supplementary Information

**Additional file 1:** Data processing details and additional results.

## Data Availability

All the source data in this work are based on the Trialtrove database, with clinical trial details derived from clinical trial publicity platforms. The datasets used and analyzed during the study are available from the corresponding author on reasonable request.

## Abbreviations

mRNA: Messenger RNA
IIT: Investigator-initiated trial
LNP: Lipid nanoparticle
LPX: Lipoplex
LPP: Lipopolyplex
DC: Dendritic cell
ICI: Immune checkpoint inhibitor
