# Supplementary material for: Clinical development of mRNA therapies against solid tumors
Source: J Hematol Oncol. 2023 Jul 18;16:75. doi: 10.1186/s13045-023-01457-x (PMC10354897; doi:10.1186/s13045-023-01457-x)
Supplement: Supplementary file 1 — Additional file 1: Data processing details and additional results. [file 13045_2023_1457_MOESM1_ESM.docx]

**Additional file** Supplementary material for

**Clinical development of mRNA therapies against solid tumors**

Dawei Wu^1†^, Lingfeng Hu^2†^, Xin Wang^1,3†^, Yue Yu^1†^, Shuo-Peng Jia^1,4^, Hui-Yao Huang^1^, Zi-Wei Li^1,5^, Jin-Feng Ma^3^, Hai-Bo Zhu^3^, Yu Tang^1^, Ning Li^1*^

*Correspondence: lining@cicams.ac.cn

^†^Dawei Wu, Lingfeng Hu, Xin Wang and Yue Yu have contributed equally to this work.

**Figures**

**Fig. S1:** Data processing flow and variables

All clinical trials registered in the Trialtrove database

as of December 31, 2021

Cancer clinical trials with mRNA agents retrieved (n = 117)

Cancer clinical trials with mRNA agents included (n = 108)

Screening with the query (Primary Tested Drug or Other Tested Drug: Therapeutic Class is Messenger RNA) AND (Primary Tested Drug or Other Tested Drug: Therapeutic Class is anticancer) AND (Therapeutic Area is Oncology)

Screening with the query [(Primary Tested Drug or Other Tested Drug: Therapeutic Class contains Messenger RNA) AND [(Primary Tested Drug or Other Tested Drug: Therapeutic Class contains anticancer) AND (Therapeutic Area contains oncology)

Exclusion of non-oncology clinical trials and clinical trials without mRNA agents (n=9)

- Trial-level: study phase, cancer type, sponsor type, sponsor country, treatment line, combination strategy and target, encoded protein
- Drug-level: delivery system, encoded protein

**Identification**

**Screening**

**Eligibility**

**Variables**

**
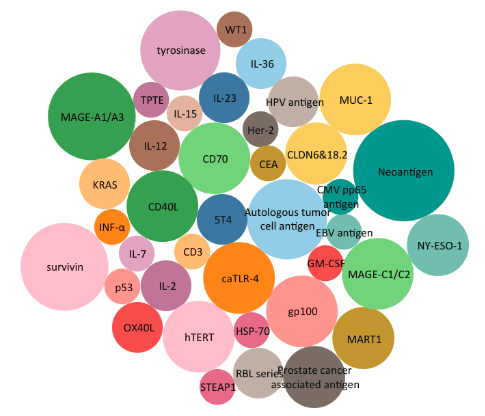
**

**b**

**a**

**Fig. S2:** The encoded proteins of anticancer mRNA therapeutics.

**a.** Distribution of coding categories; **b.** Distribution of specific encoded proteins.

**Fig. S3:** Five-year numbers of newly initiated trials for mRNA therapies encoding fix tumor antigens and personalized neoantigens

**Tables**

**Table S1:** Distribution of sponsor countries by sponsor type of clinical trials for anticancer mRNA therapeutics

| Sponsor Country | Sponsor type | | Total |
| --- | --- | --- | --- |
|  | IIT | IST |  |
| Germany | 10 | 22 | 32 |
| USA | 19 | 8 | 27 |
| Belgium | 5 | 8 | 13 |
| China | 3 | 7 | 10 |
| Korea | 0 | 6 | 6 |
| Norway | 5 | 0 | 5 |
| Switzerland | 1 | 3 | 4 |
| Netherlands | 3 | 0 | 3 |
| United Kingdom | 0 | 2 | 2 |
| Austria | 0 | 2 | 2 |
| Denmark | 2 | 0 | 2 |
| Japan | 1 | 0 | 1 |
| France | 0 | 1 | 1 |
| **Total** | **49** | **59** | **108** |

**Table S2:** Distribution of delivery systems for anticancer mRNA therapeutics

| Delivery system | | No. of agents (%) |
| --- | --- | --- |
|  |  |  |
| **Lipid-based** | | **27 (47.7%)** |
|  | LNP | 13 (22.8%) |
|  | LPX | 8 (14.0%) |
|  | LPP | 6 (10.5%) |
| **Dendritic cell** | | **16 (28.1%)** |
| **Naked RNA** | | **7 (12.3%)** |
| **Protamine-bound** | | **5 (8.8%)** |
| **Undisclosed** | | **2 (3.5%)** |
| **Total** | | **57 (100.0%)** |

**Table S3:** Preliminary clinical trial results of anticancer mRNA therapeutics

| Protocol No. | Investigational product | Indication | Combination therapy | Delivery system | Encoded proteins | Efficacy |
| --- | --- | --- | --- | --- | --- | --- |
| NCT01302496 | TriMix-DC | Advanced melanoma | Ipilimumab | DC | Immune agonists, antibodies (caTLR4, CD40L, CD70) | ORR: 38%, including 8 CRs, 7 PRs, 18 PDs. The mPFS was 6.2 months and mOS was 14.4 months^1^. |
| NCT03468244 | Individualized Neoantigen Vaccine (Shanghai Siwei) | Advanced gastrointestinal neoplasms | - | LPP | Personalized neoantigens | Data from the interim analysis showed that PFS of the 3 enrolled patients was 3.2, 3.0, and 3.0 months. OS was 9.1, 6.0, and 7.8 months, respectively^2^. |
| NCT03739931 | mRNA-2752 | Relapsed or refractory solid tumors | Monotherapy or combined with durvalumab | LNP | Cytokines (OX40L, IL-23, IL-36) | Of the 17 subjects, 1 had PR, 6 had SD, and 10 had PD^3^. |
| NCT02410733 | BNT-111 | Advanced melanoma | Monotherapy or combined with pembrolizumab | LPX | Fixed tumor antigens (NY-ESO-1, MAGE-A3, tyrosinase, TPTE) | Of the 25 subjects in the monotherapy group, 3 had PR and 7 had SD. Of the 17 subjects in the combination group, 6 had PR^4^. |
| NCT01915524 | CV-9202 | Maintenance therapy after chemotherapy in advanced NSCLC | Chemotherapy or radiotherapy | Protamine-bound | Fixed tumor antigens (NY-ESO-1, MAGE-C1, MAGE-C2, 5T4, survivin, MUC-1) | Of the 26 subjects assessed, 12 had SD and 1 had PR^5^. |
| NCT01817738 | CV-9104 | CRPC | - | Protamine-bound | Fixed tumor antigens (PAP, MUC-1) | The mOS was 35.5 months in 134 subjects of the experimental group and 33.7 months in 63 subjects of the placebo control group, with no statistical significance^6^. |
| NCT00923312 | CV-9201 | Advanced NSCLC | - | Protamine-bound | Fixed tumor antigens (NY-ESO-1, MAGE-C1/2, survivin, 5T4) | Of the 29 evaluable subjects, 9 had SD, 20 had PD. The mPFS was 5.0 months, and mOS was 10.8 months^7^. |
| NCT00831467 | CV-9103 | CRPC | - | Protamine-bound | Fixed tumor antigens (PSA, PSCA, PSMA, STEAP1) | One patient showed a confirmed PSA response. In the subgroup of 36 metastatic patients, the mOS was 31.4 months^8^. |
| NCT00693095 | Electrophoresis of shLAPM-pp65 into autologous DCs | Newly diagnosed glioblastoma | Chemotherapy | DC | Fixed tumor antigens (CMV) | In the 21 evaluable subjects, the mPFS was 12.5 months^9^. |
| NCT01446731 | mRNA transfected autologous DCs | CRPC | Chemotherapy | DC | Fixed tumor antigens (PSA) | The PFS and OS was 5.5 months and 21.9 months in the chemotherapy group, 5.7 months and 25.1 months in the combination group, respectively^10^. |
| NCT03313778 | mRNA-4157 | Advanced solid tumors | Monotherapy or combined with pembrolizumab | LNP | Personalized neoantigens | In the 10 patients with head and neck squamous cell carcinoma, ORR was 50%, including 2 CRs, 3 PRs^11^. |
| NCT00639639 | Electrophoresis of shLAPM-pp65 into autologous DCs | Newly diagnosed glioblastoma | Chemotherapy | DC | Fixed tumor antigens (CMV) | In the 13 subjects, the mPFS was 15.4 months and mOS was 20.6 months^12^. |
| NCT00626483 | Electrophoresis of shLAPM-pp65 into autologous DCs | Newly diagnosed glioblastoma | Basiliximab | DC | Fixed tumor antigens (CMV) | In the 28 subjects, the mPFS was 7.7 months^13^. |
| NCT03289962 | BNT-122 | Advanced solid tumors | Atezolizumab | LNP | Personalized neoantigens | In the 108 evaluable subjects, the ORR was 8%. One colorectal cancer patient had CR, and 53 subjects had SD^14^. |
| NCT01582672 | Rocapuldencel-T | First-line therapy of advanced renal cell carcinoma | Sunitinib | DC | Personalized neoantigens | The mPFS was 6.0 and 7.8 months, mOS was 27.7 and 32.4 months for the combination group and the sunitinib group, respectively^15^. |

DC: Dendritic cell, ORR: Objective response rate; CR: Complete response, PR: Partial response, PD: Progressed disease, PFS: Progression free survival, OS: Overall survival, LPP: Lipopolyplex, LNP: Lipid nanoparticle, SD: Stable disease, LPX: Lipoplex, NSCLC: Non-small-cell lung cancer, CRPC: Castration resistant prostate cancer, PSA: Prostate specific antigen.

**Reference of Table S2:**

1. De Keersmaecker B, Claerhout S, Carrasco J, et al. TriMix and tumor antigen mRNA electroporated dendritic cell vaccination plus ipilimumab: link between T-cell activation and clinical responses in advanced melanoma. *Journal for immunotherapy of cancer*, 2020, 8(1).
2. Zhan X, Wang B, Wang Y, et al. Phase I trial of personalized mRNA vaccine encoding neoantigen in patients with advanced digestive system neoplasms. *Journal of Clinical Oncology*, 2020, 38(15_suppl): e15269-e.
3. Patel M R, Bauer T M, Jimeno A, et al. A phase I study of mRNA-2752, a lipid nanoparticle encapsulating mRNAs encoding human OX40L, IL-23, and IL-36γ, for intratumoral (iTu) injection alone and in combination with durvalumab . *Journal of Clinical Oncology*, 2020, 38(15_suppl): 3092-.
4. Sahin U, Oehm P, Derhovanessian E, et al. An RNA vaccine drives immunity in checkpoint-inhibitor-treated melanoma. *Nature*, 2020, 585(7823): 107-112.
5. Papachristofilou A, Hipp M M, Klinkhardt U, et al. Phase Ib evaluation of a self-adjuvanted protamine formulated mRNA-based active cancer immunotherapy, BI1361849 (CV9202), combined with local radiation treatment in patients with stage IV non-small cell lung cancer. *Journal for immunotherapy of cancer*, 2019, 7: 1-14.
6. Stenzl A, Feyerabend S, Kübler, H, et al. 1155PResults of an open label randomized phase II trial of CV9104, an mRNA-based multivalent cancer immunotherapy in patients (pts) with intermediate or high risk localized prostate cancer (PC) undergoing radical prostatectomy (RPE). *Annals of Oncology*, 2017, 28(suppl_5).
7. Sebastian M, Schröder A, Scheel B, et al. A phase I/IIa study of the mRNA-based cancer immunotherapy CV9201 in patients with stage IIIB/IV non-small cell lung cancer. *Cancer Immunology, Immunotherapy*, 2019, 68: 799-812.
8. Kübler H, Scheel B, Gnad-Vogt U, et al. Self-adjuvanted mRNA vaccination in advanced prostate cancer patients: a first-in-man phase I/IIa study. *Journal for immunotherapy of cancer*, 2015, 3(1): 1-14.
9. Mitchell D, Archer G, Bigner D, et al. Efficacy and immunologic effects of RNA-pulsed dendritic cell vaccines targeting human cytomegalovirus antigens in patients with glioblastoma. *Cancer Research*, 2008, 68(5):LB-73-LB-73.
10. Kongsted P, Borch T H, Ellebaek E, et al. Dendritic cell vaccination in combination with docetaxel for patients with metastatic castration-resistant prostate cancer: A randomized phase II study[J]. Cytotherapy, 2017, 19(4): 500-513.
11. Positive preliminary Phase 1 data reported in PCV and IL-12 [EB/OL].2021,from https://investors.modernatx.com/static-files/36d85cf5-365b-4c5c-a8e5-017e973bcdb9
12. Batich K A, Mitchell D A, Healy P, et al. Once, Twice, Three Times a Finding: Reproducibility of Dendritic Cell Vaccine Trials Targeting Cytomegalovirus in Glioblastoma Reproducibility of Dendritic Cell Vaccines in Glioblastoma. *Clinical Cancer Research*, 2020, 26(20): 5297-5303.
13. Vlahovic G, Archer G E, Reap E, et al. Phase I trial of combination of antitumor immunotherapy targeted against cytomegalovirus (CMV) plus regulatory T-cell inhibition in patients with newly-diagnosed glioblastoma multiforme (GBM). *Journal of Clinical Oncology*, 2016, 34(15_suppl): e13518-e.
14. Braiteh F, LoRusso P, Balmanoukian A, et al. A phase Ia study to evaluate RO7198457, an individualized Neoantigen Specific immunoTherapy (iNeST), in patients with locally advanced or metastatic solid tumors[C]//*Proceedings of the Annual Meeting of the American Association for Cancer Research*. 2020, 2020: 27-28.
15. Figlin R A, Tannir N M, Uzzo R G, et al. Results of the ADAPT phase 3 study of rocapuldencel-T in combination with sunitinib as first-line therapy in patients with metastatic renal cell carcinoma. *Clin Cancer Res*, 2020, 26(10): 2327-36.

**Table S4:** The combination strategies of clinical trials for mRNA therapeutics

| Combination strategy | | No. of trials |
| --- | --- | --- |
|  |  |  |
| **Single-agent only** | | **36** |
| **Combination*** | | **59** |
|  | Chemotherapy | 20 |
|  | Targeted therapy | 6 |
|  | **Immunotherapy** | **44** |
|  | Checkpoint inhibitor | 25 |
|  | Other immuno-modulator | 9 |
|  | Cell therapy | 7 |
|  | Cancer vaccine | 3 |
|  | Radiotherapy | 3 |
| **Undisclosed** | | **13** |

*Multiple combination strategies may be involved in the same clinical trial.
